# Return of large fin whale feeding aggregations to historical whaling grounds in the Southern Ocean

**DOI:** 10.1038/s41598-022-13798-7

**Published:** 2022-07-07

**Authors:** Helena Herr, Sacha Viquerat, Fredi Devas, Abigail Lees, Lucy Wells, Bertie Gregory, Ted Giffords, Dan Beecham, Bettina Meyer

**Affiliations:** 1 Institute of Marine Ecosystem and Fishery Science, Center for Earth System Research and Sustainability, University of Hamburg, Große Elbstraße 133, 22767 Hamburg, Germany; 2 Alfred Wegener Institute Helmholtz Centre for Polar and Marine Research, Am Handelshafen 12, 27570 Bremerhaven, Germany; 3 BBC Studios, Natural History Unit, Bridgewaterhouse, Counterslip, Bristol, UK; 4 Institute for Chemistry and Biology of the Marine Environment, Carl von Ossietzky University of Oldenburg, Carl-von-Ossietzky-Straße 9-11, 26111 Oldenburg, Germany; 5 Helmholtz Institute for Functional Marine Biodiversity at the University of Oldenburg (HIFMB), Ammerländer Heerstraße 231, 26129 Oldenburg, Germany

**Keywords:** Ecology, Behavioural ecology, Conservation biology, Ecosystem ecology, Population dynamics, Marine biology

## Abstract

Fin whales (*Balaenoptera physalus quoyi*) of the Southern Hemisphere were brought to near extinction by twentieth century industrial whaling. For decades, they had all but disappeared from previously highly frequented feeding grounds in Antarctic waters. Our dedicated surveys now confirm their return to ancestral feeding grounds, gathering at the Antarctic Peninsula in large aggregations to feed. We report on the results of an abundance survey and present the first scientific documentation of large fin whale feeding aggregations at Elephant Island, Antarctica, including the first ever video documentation. We interpret high densities, re-establishment of historical behaviours and the return to ancestral feeding grounds as signs for a recovering population. Recovery of a large whale population has the potential to augment primary productivity at their feeding grounds through the effects of nutrient recycling, known as 'the whale pump'. The recovery of fin whales in that area could thus restore ecosystem functions crucial for atmospheric carbon regulation in the world's most important ocean region for the uptake of anthropogenic CO_2_.

## Introduction

Fin whales (*Balaenoptera physalus quoyi*) of the Southern Hemisphere were brought to near extinction by twentieth century industrial whaling^1^. More than 700,000 individuals were killed^2^ between 1904, when intensive commercial whaling began in the Southern Ocean, and 1976, when the catch quota of fin whales was set to zero, 10 years before the moratorium on whaling^3,4^. It has been estimated that by then the population had been reduced to 1–2% of its pre-exploitation size of around 325,000 animals in the early twentieth century^2,5,6^. Major whaling for fin whales took place at their feeding grounds at the northern tip of the Antarctic Peninsula^7^. After the end of commercial whaling, cetacean surveys conducted under the auspices of the International Whaling Commission (IWC) between 1978 and 2004 (IDCR/SOWER surveys), reported very few fin whales in that region^8^. They had seemingly vanished from those historical feeding grounds. About 40 years later, our surveys now confirm a return of the whales to their ancestral feeding grounds in high numbers, forming large feeding aggregations.

In the post-whaling era, the International Whaling Commission’s (IWC) International Decade of Cetacean Research (IDCR) and Southern Ocean Whale Ecosystem Research (SOWER) cruise programmes, carried out in three circumpolar sets of surveys between 1978 and 2004, provided the only comprehensive information on fin whale abundance in the Southern Hemisphere. Based on IDCR/SOWER data from surveys between 1991 and 1998, circumpolar fin whale abundance south of 60°S was estimated at 5445 (95% CI 2000–14,500)^8^. However, since the surveys did not cover the full latitudinal distribution of fin whales and an unknown proportion of the population will have ranged north of 60°S even in summer, this estimate almost certainly represents an underestimate. For the Scotia Arc and Antarctic Peninsula region, fin whale abundance was last estimated at 4672 (CV 42.37) based on data from the CCAMLR/SOWER survey conducted in 2000^9^. Globally, fin whales are listed as 'vulnerable' on the IUCN Red List of Threatened Species^10^. Their status was changed from 'endangered' to 'vulnerable' in 2018 based on projections of the global mature population size. It was however noted that global population estimates were associated with much uncertainty due to lack of data especially from mid-latitudes in the Southern Hemisphere^10^.

Since the 2000s, observations of fin whales from the Antarctic Peninsula region have been increasing. First, surveys using platforms of opportunity traveling between South America and the Antarctic Peninsula in 2001 and 2002 indicated considerable densities of fin whales in the offshore waters running parallel to the Antarctic Peninsula. Shortly after, Santora et al.^11^ suggested hotspots of fin whale occurrence in the Southern Drake Passage and around Elephant Island^12^ based on high encounter rates recorded during krill surveys between 2003 and 2011. In 2012, an opportunistic observation of an aggregation of more than 100 animals was reported^13,14^. A year later, an aerial cetacean survey around the South Shetland Islands provided an abundance estimate of 4898 (95% CI 2221–7575) fin whales (survey area: ~ 42,000 km^2^; density = 0.117; 95% CI 0.053–0.181 individuals/km^2^), with observations of fin whales feeding in groups of up to 70 animals^15^. Lastly, in 2016, a shipboard cetacean survey reported high fin whale densities around Elephant Island (0.0268 ± 0.0183 individuals/km^2^) and the South Orkney Islands (0.0588 ± 0.0381 individuals/km^2^)^16^. These consistently high numbers were our motivation for a dedicated assessment. We therefore conducted two surveys (a shipboard survey and a vessel-supported helicopter survey) around the northern tip of the Antarctic Peninsula to estimate fin whale abundance and to investigate the new phenomenon of fin whale feeding aggregations.

In this paper, we report on the results of an abundance survey and present the first documentation of fin whale feeding aggregations. We discuss the ecological implications of the recovery of a large baleen whale species and its return to ancestral feeding grounds against the background of ecosystem services provided by whales.

## Methods and fieldwork

### Data collection

Data were collected during two expeditions to the Antarctic Peninsula in 2018 and 2019.

During the multidisciplinary research expedition PS112 (18 March–5 May 2018^17^) of the German research ice breaker *Polarstern*^18^, we conducted a vessel-supported helicopter survey [using the on-board helicopter (BO-105)] to estimate abundance of fin whales along the northern tip of the Antarctic Peninsula. Data collection followed line transect distance sampling methodology^19^ and an adaptive *ad-hoc* survey design^15,20^. Owing to logistics of a multidisciplinary research cruise, where the ship simultaneously caters to the needs of several research projects on board^21^, it was impossible to follow a pre-designed fixed survey scheme. Instead, aerial transects were placed around the current position of the ship, aiming at an adequate overall coverage of the survey area and applying basic principles of good survey design following Buckland et al.^19^ (i.e., arbitrary orientation and placement of transects with respect to whale distribution). Based on this *ad-hoc* method (described in more detail in Herr et al.^15^), our survey was planned in anticipation of model-based abundance estimation^22–24^ rather than conventional design-based analysis^24,25^. Flight altitude was 600 ft at a survey speed of 80–90 knots. Two experienced observers (the same throughout the whole survey) seated in the front and back left seats of the helicopter collected sighting data. The front observer covered the area directly below the helicopter, i.e. the transect line and up to 80 m to the left, through the bottom front window. The back observer covered the remaining area to the left of the transect line up to the horizon. Together, both observers provided full coverage of the left side of the transect line and were treated as one observer during analysis. To avoid potential duplication of sightings, continuous communication was maintained via intercom. Coverage of only one side of the transect was accounted for during analysis (i.e. detection function modelling, see below). All data were entered directly into a computer running dedicated data collection software (VORaudio, designed by Lex Hiby and Phil Lovell), continuously storing GPS data obtained via a GPS device (Garmin 72H). Sighting conditions were judged by the observers and information entered with every change therein. The following two measurements were used: sea state in the Beaufort scale and 'subjective sighting conditions', i.e. the observers' evaluation of the chances to detect an available large whale (a compound variable taking the levels 'good', 'moderate', and 'poor'). For each sighting, the species, declination angle from observer to animal, and group size were noted. Declination angles were measured using inclinometers and were used to calculate distances of sightings to the transect line.

Survey flights were accompanied by a camera operator to film fin whale encounters, and particularly, feeding events. Additional sightings of fin whale aggregations were documented opportunistically during ship transit, logging position and group size.

In 2019, the *Pelagic Australis* expedition revisited the survey area of the PS112 expedition. This expedition was dedicated to media purposes and the documentation of fin whale feeding aggregations, therefore a line transect survey was not conducted. Fin whale aggregations were explicitly searched for and encounters were recorded and filmed.

### Video documentation

Video imagery in 2018 was collected from the helicopter, from the *RV Polarstern* deck and using drones. In 2019, videos were made from drones and an inflatable powerboat, both deployed from the *Pelagic Australis*. A stabilised camera system was attached to the helicopter, using a RED Helium 8 K camera with Canon CN20 (50–1000 mm) lens inside a GSS gyro-stabilised system to film during helicopter flights. The same system was used to film from the deck of the ship. If feeding aggregations were encountered during ship transit, drones (DJI Phantom 4 and Inspire II equipped with a Zenmuse X5S camera) were launched to collect aerial imagery.

### Analyses

We used all sighting records of fin whales (not including any aggregations) collected during helicopter survey effort in a distance sampling analysis for abundance estimation^19,23,25^. First, we modelled a detection function to account for the effects of distance and other covariates on the detection probability of whale groups. We used the R software package ‘Distance’^26^ for a multiple covariate distance sampling (MCDS) analysis^27^, assuming that the probability of detection on the track line was 1 (i.e., 100%). Mean group size was estimated via the regression method^19^ to account for effects of group size bias on detectability. In theory, at large distances large group sizes are more likely to be detected than small groups or single animals. Therefore, this bias can be accounted for by estimating a correction based on the regression of group sizes with distance. Sighting data were manually right-truncated at 1750 m after visual inspection of the distribution of sightings to exclude outliers at large distances. We used sea state and subjective sighting conditions (i.e., 'good', moderate' and 'poor') as potential covariates in a half-normal and hazard-rate detection function model, including a cosine adjustment series of order 2 for the half-normal and no adjustment for the hazard rate models. Model selection was based on Akaike’s information criterion (AIC,^28^) and the model’s capability to accurately capture the number of sightings near the transect line p_0_ and Goodness of fit as expressed by the Cramér van Mises test statistic.

For the density surface model, we aggregated effort and the number of fin whale groups and individuals to segments of 5 km length along the transect lines (sometimes resulting in segments < 5 km at the end of transects or effort and discarding all segments with a total length < 1 km) and calculated the effectively covered area within each segment as:

1 $${A}_{seg}=esw\times {L}_{seg}$$

With ${A}_{seg}$ the effectively covered area [km^2^] along segment *seg*, $esw$ the effective half strip width [km] based on the detection function model and ${L}_{seg}$ the effort [km] along segment *seg*.

We then used the ‘mgcv’ package^29,30^ to fit an additive model of the observed fin whale groups per segment, off set by the log of the effectively searched area per segment, to a smoothed interaction of *x* and *y* (projected longitude and latitude values of segment midpoints, respectively) and combinations of *x* and *y* with water depth (IBCSO v2^31^) and derived properties TPI (topographic position index)^32^, TRI (terrain ruggedness index)^32^, slope and aspect. TPI, TRI, slope and aspect were calculated from the depth raster using the ‘raster’ package^33^. Segment covariates were extracted along each segment and averaged for the whole segment. Additional covariates tested in the models were the calculated distance from the segment midpoints to the shelf break (as defined in Herr et al.^20^) and to the nearest coastline. Since our main interest was a simple and robust snapshot of abundance and distribution rather than an ecological model describing drivers of distribution, we included variables in addition to *x* and *y* only to test if they improved the simplest model containing only *x* and *y*^34^. Since this was not the case, we did not include any interactions between the covariates and no testing for correlation between covariates was needed.

We used a thin plate smoother for all terms and an (auto starting) Tweedie error distribution for the model residuals^30,35^. Final model selection was based on AIC^28^, their generalised cross validation score (GCV^36^) and deviance explained. In case of similar model performance, we opted for the simpler model, i.e. the model with fewer covariates.

**Figure 1:**
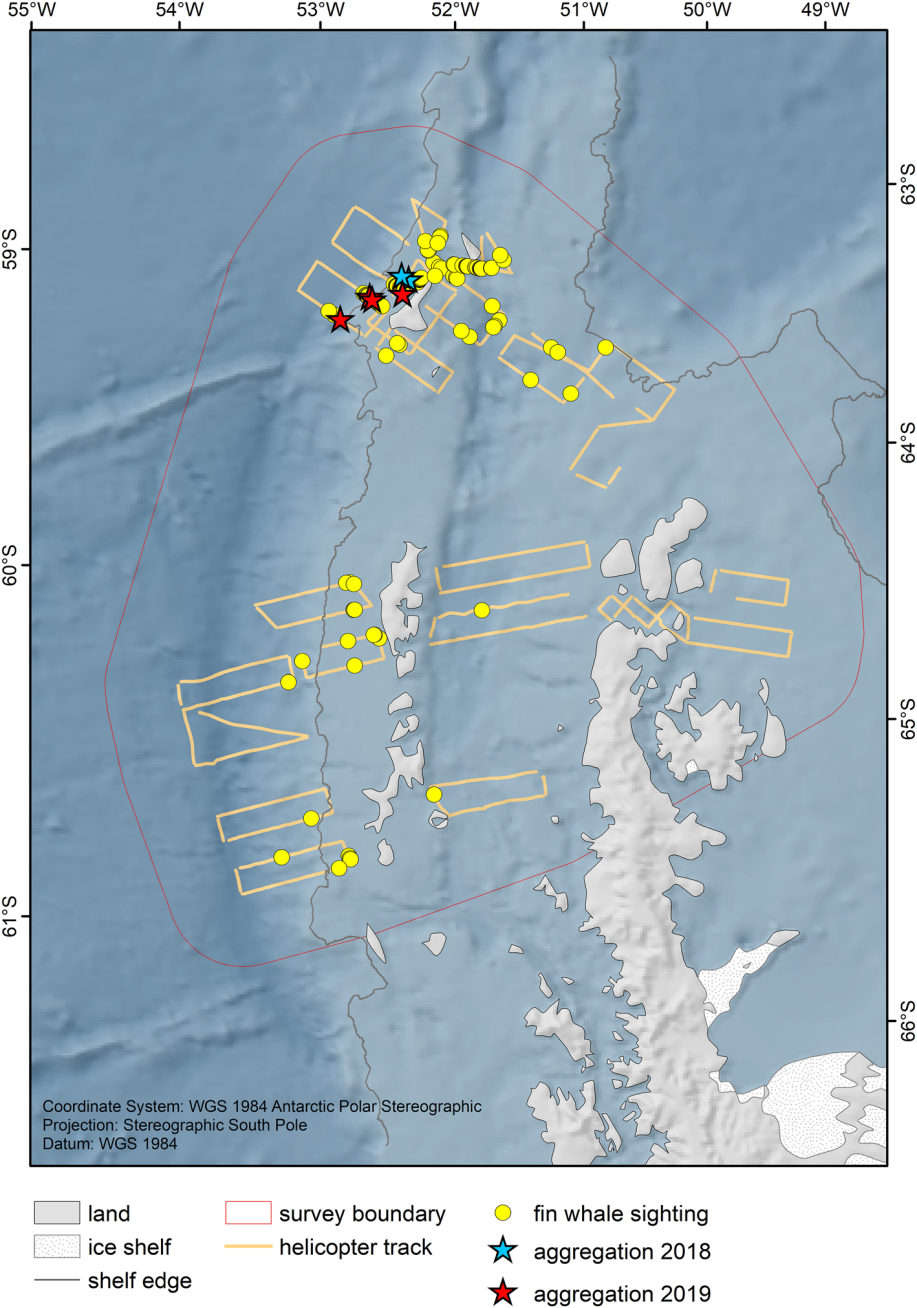
Survey effort and fin whale sightings. Representation of transect lines covered by the aerial survey during *RV Polarstern* expedition PS112. Fin whale sightings recorded on-effort during the aerial survey are indicated as yellow dots. Positions of fin whale aggregations (stars) comprise sightings collected during both expeditions. The map was composed using ESRI ArcGIS 10.6 (https://www.esri.com/en-us/arcgis/products/arcgis-desktop/resources).

We covered the surveyed area (i.e. the area within the survey boundary, Fig. 1) with a grid of 2.5 × 2.5 km cells, attributed with the same covariates as used in the density surface model, and predicted the number of fin whale groups per grid cell across this area. We assessed the CVs associated with the predictions in every cell and discarded cells with CVs ≥ 100, limiting the spatial extent of the prediction area to areas supported by sufficient data coverage and avoiding extrapolation beyond reasonable boundaries. The remaining area served as the prediction area for which we estimated fin whale abundance based on the model. To translate group density results to individual abundance we multiplied the number of groups by the group size of fin whales estimated from the regression of original sighting records across all observed distances. The total abundance was based on the sum of abundance of all cells within the area. We used the standard error as reported by the model for the calculation of confidence intervals across the whole study area. All analyses were done in R version 3.6.1 (R Core Team 2019). Imagery was used to document feeding aggregations and behaviour, and to aid group size estimation of large feeding aggregations. We defined feeding behaviour as a display of lunges, repetitive and consecutive diving behaviours and expanded buccal cavities at surfacing. We use the term aggregation to describe groups of 15 or more individual whales estimated to be within five body lengths of their nearest neighbour.

## Results

### Fin whale records and group sizes

**Figure 2:**
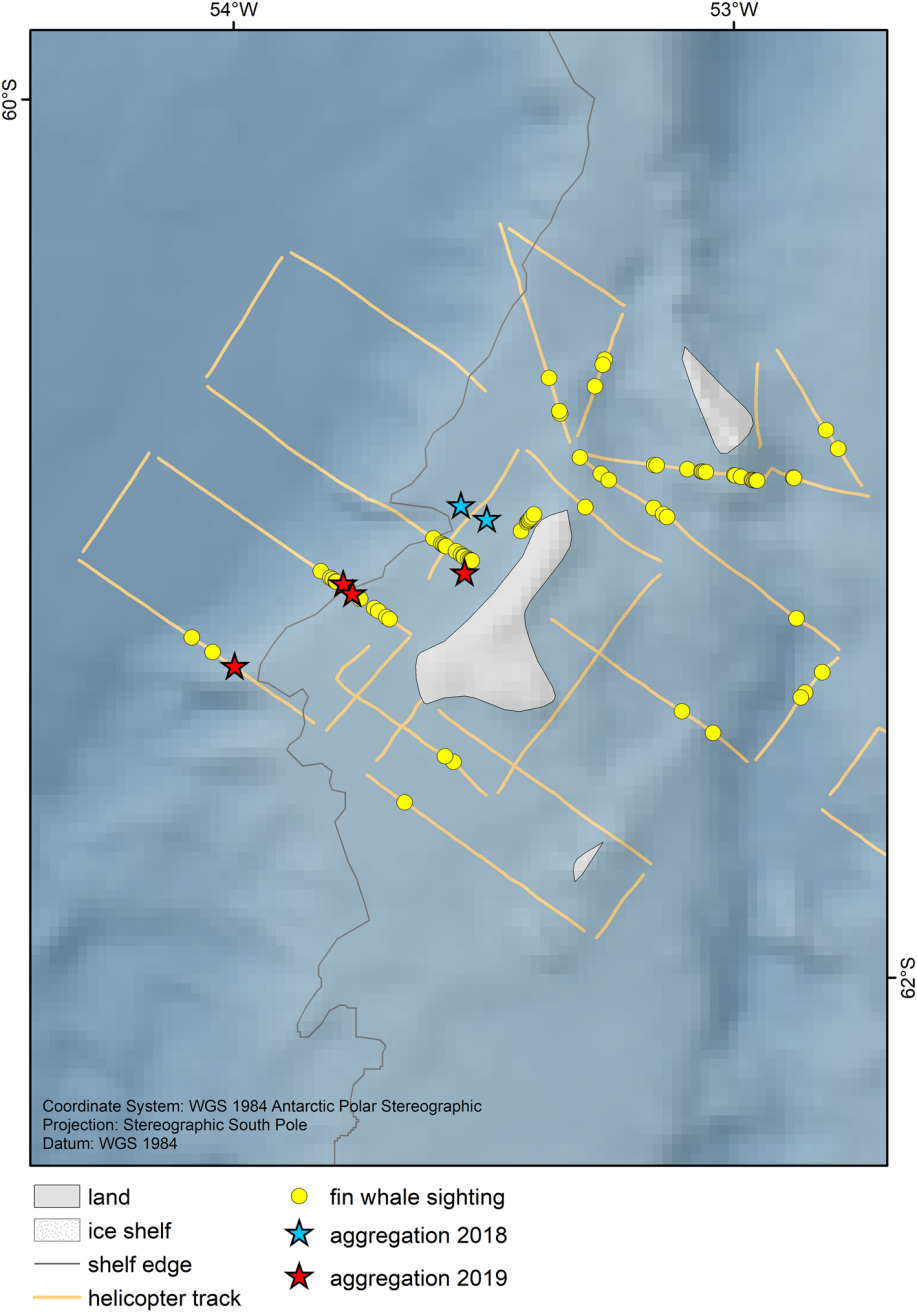
Close up view of survey effort and sightings around Elephant Island. All aggregations during both expeditions were recorded at the northern coast of Elephant Island. The map was composed using ESRI ArcGIS 10.6 (https://www.esri.com/en-us/arcgis/products/arcgis-desktop/resources).

**Figure 3:**
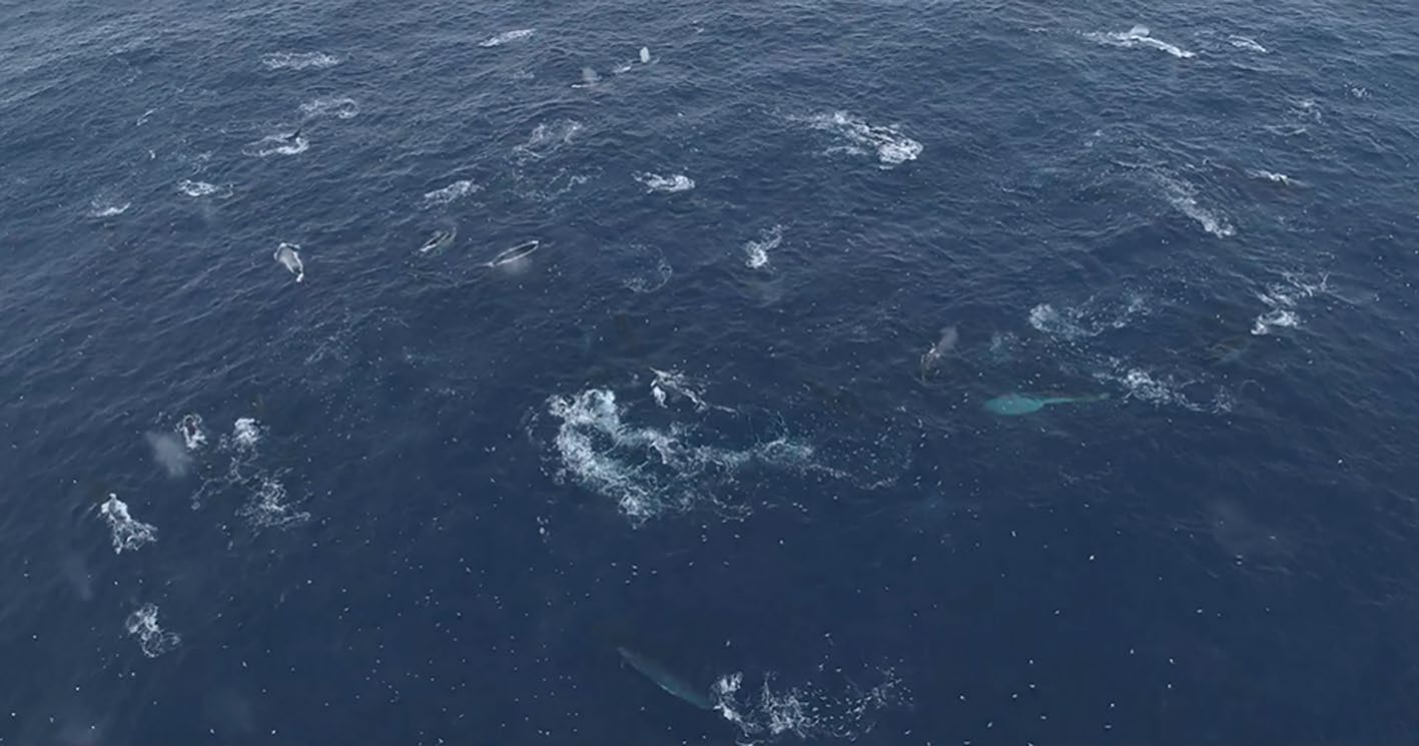
Fin whale feeding aggregation. Aerial view on a section of the active feeding aggregation of ~ 70 fin whales encountered during ship transit on *RV Polarstern* expedition PS112 in 2018, filmed by drone. ©BBC (OSM video 2).

**Figure 4:**
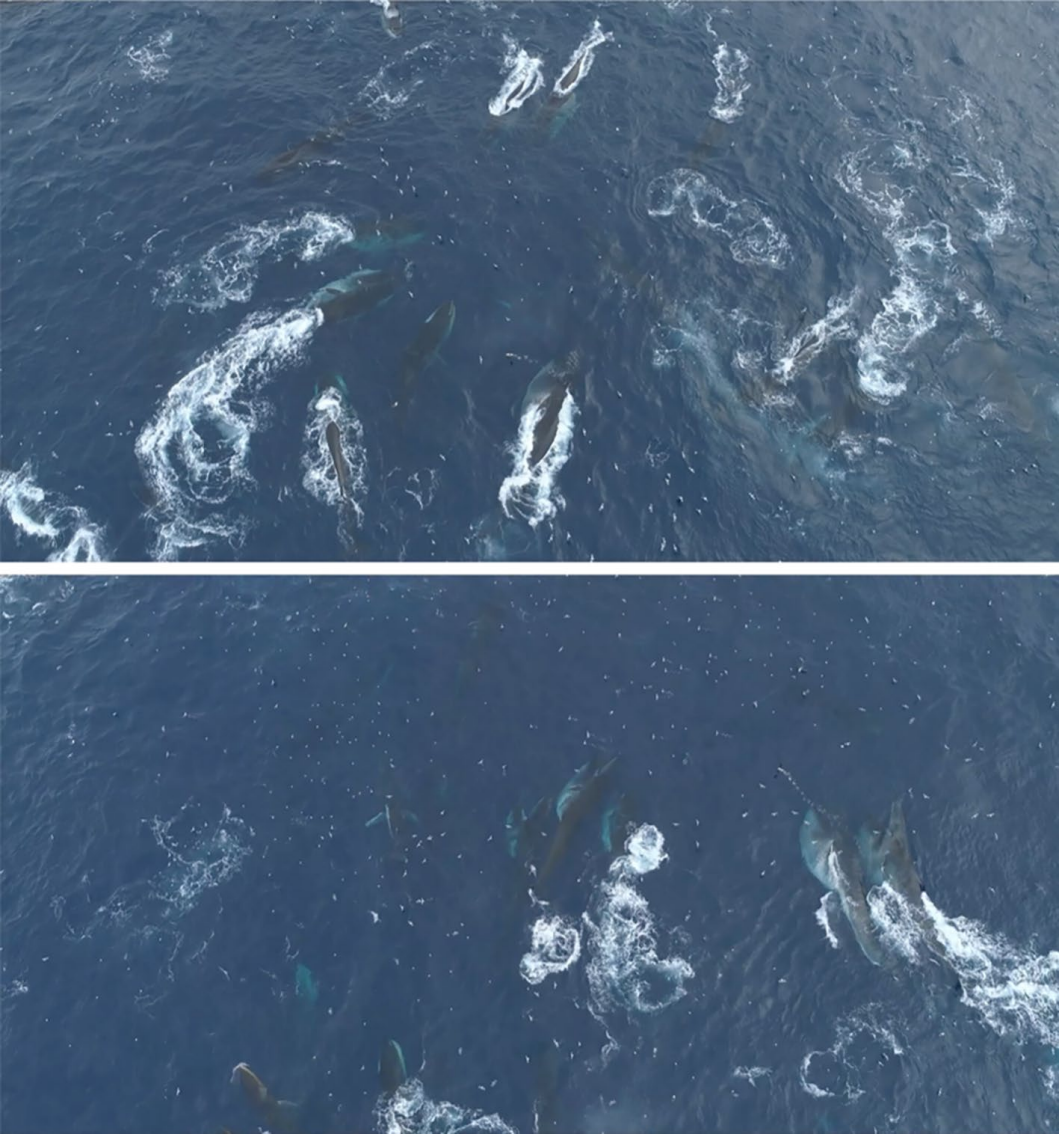
Close-up sections of the active feeding aggregation of ~ 70 fin whales. This aggregation was encountered during ship transit on *RV Polarstern* expedition PS112 in 2018 and filmed by drone. Fin whales are side-lunge feeding together at the surface. In the bottom panel a single humpback whale feeding with the fin whales is visible. ©BBC (OSM video 3).

**Figure 5:**
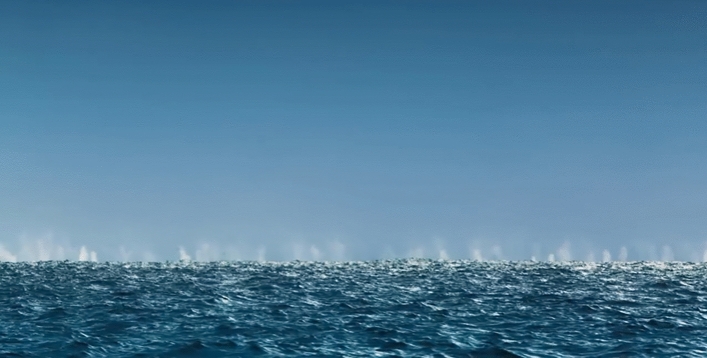
Fin whale feeding aggregation at a distance. The horizon is covered by blows of a feeding aggregation numbering approximately 150 fin whales. ©BBC (OSM video 4).

During the aerial survey of expedition PS112, we completed 22 survey flights between 23rd March and 24th April 2018, covering 3251 km of track lines during 26.3 h of search effort. We recorded 100 groups of fin whales on effort (Fig. 1), with group sizes ranging from 1–4 individuals and a mean group size of 1.28 ± 0.04 animals. No aggregations were encountered during aerial survey effort and thus no records of aggregations were included in the distance analysis. A small aggregation of 15 animals was encountered off-effort (i.e., during helicopter transit to a transect), with fin whales feeding together with Antarctic fur seals *(Arctocephalus gazella)* and chinstrap penguins *(Pygoscelis antarcticus)* (Table 1). During ship transit, two aggregations of approximately 50 and 70 animals respectively were encountered (Fig. 2, Table 1). The first one was encountered in a situation interpreted as 'post-feeding' (Online Supplementary Material (OSM) Video 1) with a high density of animals in an active behavioural state, but not feeding. The second aggregation was encountered with intense feeding activity ongoing (Fig. 3, OSM Video 2). Drone footage provided aerial close-up imagery of feeding behaviour (Fig. 4, OSM Video 3). During the Pelagic Australis expedition in 2019, five fin whale aggregations were recorded at Elephant Island (Table 1, Figs. 1, 2), with the largest two counting approximately 150 animals (Fig. 5, OSM Video 4).

**Table 1:** Aggregations recorded during RV Polarstern expedition PS112 in 2018 and the Pelagic Australis expedition in 2019.

Date	Expedition	Number of animals	Location (Lat/Lon)	Comments
07 April 2018	PS112	15	S 61.078 W 054.739	+ fur seals, chinstrap penguins
07 April 2018	PS112	50	S 61.018 W 054.842	
24 April 2018	PS112	70	S 60.970 W 054.855	+ 1 humpback whale
18 March 2019	P. Australis	30	S 60.813 W 054.855	
19 March 2019	P. Australis	50	S 60.884 W 055.303	
20 March 2019	P. Australis	150	S 60.906 W 055.313	
21 March 2019	P. Australis	30	S 61.039 W 055.036	+ 2 humpback whales
23 March 2019	P. Australis	150	S 61.039 W 055.036	

Group size estimates of larger aggregations are best estimates based on counts in the field and evaluation of footage, as exact counting of individuals was not possible.

### Density and abundance estimation

**Figure 6:**
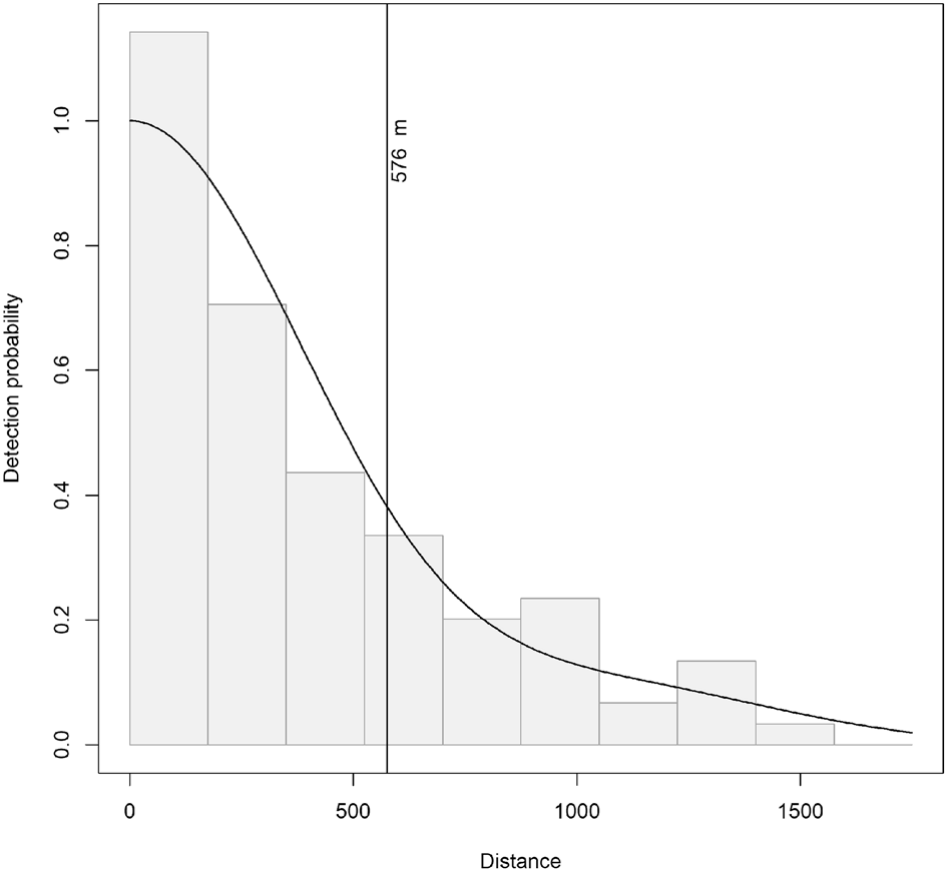
Detection function for selected model c1. The detection function chosen as the best model was a half-normal key function using a cosine series adjustment of order 2 and no additional sighting covariates. Effective strip width was estimated at 576 m.

Sighting data were truncated at a distance of 1750 m. All models using the hazard-rate key were deemed not adequate for the present data, because visual inspection of the histograms (with the overlaid detection probability curve from the hazard rate models) indicated a poor fit at distances near zero, and were therefore not considered as final models. The remaining half-normal models all achieved very similar p_0_-values (i.e., similar results for detection probability). From these, detection function model c1 using the half normal key with cosine adjustment and no additional covariates was chosen as the best model based on AIC values, Cramér von Mises *p* values and the high accuracy for p_0_ (Table 2, Fig. 6). Interactions of covariates were not tested due to insufficient sample size at factor combinations (100 sightings overall). The inclusion of covariates did not improve the detection functions. The effective half strip width was estimated at 576 m.

**Table 2:** Detection functions tested.

Key	Adj	Model	Covariates	CvM	p0 ± SE	AIC	ΔAIC
Hazard-rate	–	h2	Seastate	0.1256	0.055 ± 0.0167	1365.86	0
		h1	–	0.0897	0.1041 ± 0.0255	1371.11	5.26
		h3	Subj	0.0959	0.1033 ± 0.0262	1375.05	9.19
Half-normal	Cosine	c1	–	0.067	0.3292 ± 0.0284	1378.06	12.2
		c3	Subj	0.0622	0.326 ± 0.0297	1380.14	14.28
		c2	Seastate	0.0629	0.3288 ± 0.0284	1381.61	15.75
	–	m1	–	0.0055	0.4008 ± 0.0261	1382.52	16.66
		m3	Subj	0.0068	0.3911 ± 0.0301	1383.93	18.08
		m2	Seastate	0.0076	0.3933 ± 0.0279	1385.15	19.29

Key: key function used for the detection function model; adj: adjustment series for the function (if any); model: model name as referred to in the text; covariates: the covariates used (in addition to distance to the transect line); CvM: Cramér van Mises p-value for goodness of fit; p0: estimated average detectability of animals on the transect line (including standard error); AIC: Akaike information criterion; ΔAIC: difference in AIC (compared to model with lowest AIC).

**Figure 7:**
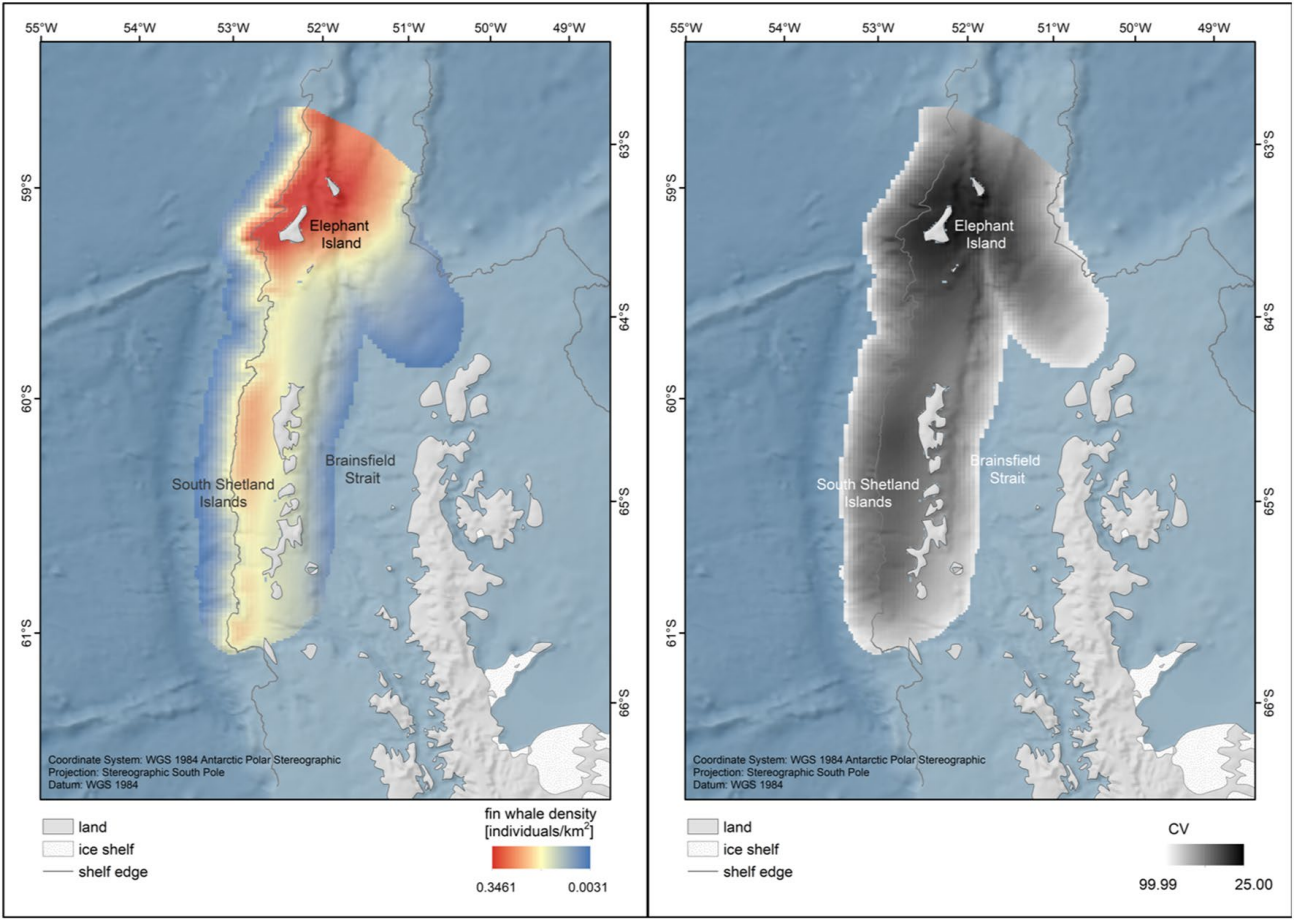
Fin whale distribution (left) and associated CVs (right) based on aerial survey data from *RV* *Polarstern* expedition PS112. Fin whale distribution as predicted by model g8 including a smooth of *x* and *y* and the distance to the shelf break as covariates. The spatial extent of the prediction was confined to only contain predictions with CVs < 100. The maps were composed using ESRI ArcGIS 10.6 (https://www.esri.com/en-us/arcgis/products/arcgis-desktop/resources).

Based on the assessment of AIC, GCV and deviance explained, we chose model g8 (including a spatial smoother across *x* and *y* and the distance to the shelf break as covariates; Table 3) as the best additive model. Model g8 achieved the lowest AIC and GCV values of all models, but was not the model with the highest deviance explained (i.e., not the model with the lowest deviance from the saturated model). While the model with the lowest deviance will certainly represent the sample data better than any other model, it may not generalise well (due to overfitting) and therefore does not guarantee small deviance on independent data. Since differences in deviance between the models were marginal, we chose the model with best AIC and GCV performance (model g8) for our prediction. Using model g8 we predicted fin whale densities over the full extent of the survey boundary (for comparison, prediction performance by all models is shown in OSM, Figure S1). The restriction to predictions associated with a CV < 100 resulted in a prediction area of 92,819 km^2^, discarding the outer margins of the survey boundary. For this area, the density surface model g8 estimated fin whale abundance at 7909 (95% CI 1047–15,743) animals, corresponding to an average density of 0.085 (95% CI 0.0113–0.1696) animals/km^2^ (Table 4). The predicted distribution of fin whales showed three centres of concentration along the shelf break west of the Antarctic Peninsula. Particularly high densities were predicted around Elephant Island (Fig. 7). In this hotspot area of 17,038 km^2^, abundance was predicted at 3618 (888–6525) animals, corresponding to an average density of 0.2123 (0.0521–0.383) animals/km^2^ (Table 4). Abundance estimates represent minimum estimates since no correction for availability or perception bias could be applied.

**Table 3:** Models tested for abundance estimation.

Model	Covariates	AIC	GCV	ΔAIC	ΔGCV	R^2^	Deviance explained (%)
*g8*	*s(x, y)* + *s(dist2shelf)*	*890.25*	*265.03*	*0*	*0*	*0.1*	*38*
g2	s(x, y) + s(depth)	891.11	265.06	0.86	0.03	0.13	39.6
g1	s(x, y)	893.95	268.99	3.7	3.95	0.13	41
g7	s(x, y) + s(dist2coast)	894.38	268.25	4.12	3.21	0.13	40
g6	s(x, y) + s(roughness)	895.57	269.75	5.32	4.72	0.13	41.48
g4	s(x, y) + s(TRI)	895.63	269.93	5.38	4.9	0.13	41.32
g3	s(x, y) + s(TPI)	895.68	270.02	5.43	4.98	0.13	41.15
g5	s(x, y) + s(aspect)	895.97	268.36	5.72	3.33	0.18	44.99

Model: model name as used throughout the manuscript (model in italics indicates selected model); covariates: the covariates used in the gam (the s indicates thin-plate smoothing terms); AIC: Akaike Information Criterion; GCV: Generalised Cross Validation Score; ΔAIC: difference in AIC (compared to model with lowest AIC); ΔGCV: difference in GCV (compared to model with lowest GCV); R^2^: pseudo R square measure of model fit; Deviance explained: alternative metric for goodness of fit.

**Table 4:** Predicted abundance and density of fin whales in the prediction area (i.e., the area for which predictions were associated with a CV < 100), and for the hotspot around Elephant Island.

	Area (km^2^)	N (95% CI)	D_groups_ (groups/km^2^) (95% CI)	D_ind_ (individuals/km^2^) (95% CI)
Prediction area	92,819	7909 (1047–15,743)	0.0107 (0.0014–0.0213)	0.0852 (0.0113–0.1696)
Elephant Island hotspot	17,038	3618 (888–6525)	0.0266 (0.0065–0.048)	0.2123 (0.0521–0.383)

N = abundance, D = density. 95% confidence intervals given in brackets. Predictions are based on model g8.

### Behavioural observations

The behaviour in aggregations was dominated by feeding, with animals lunging with mouths agape, tight turning and repeated strong vertical diving, resembling a 'feeding frenzy'^37–39^. Surfacing animals displayed expanded buccal cavities (Fig. 4, OSM videos 2 and 3). Although no prey sampling could be carried out within the tightly packed feeding aggregations, krill could be observed at the surface, and echosounder data recorded at the time of the feeding aggregation during transit of PS112 indicated dense *Euphausia superba* presence in the area (personal observation B. Meyer)^17^. Presence of other krill predator species (Antarctic fur seals and several species of birds mainly of the family *Procellariidae*) clearly pointed to availability of prey in the upper water column. On one occasion during PS112 a single humpback whale (*Megaptera novaeangliae*) was associated with a large feeding aggregation of fin whales (Fig. 4, OSM video 3), during the *Pelagic Australis* expedition, two humpback whales were observed in a fin whale feeding aggregation.

## Discussion

### Feeding aggregations

The feeding aggregations documented in our study are among the largest ever reported for baleen whales in scientific literature. Similar in size are only the so called 'super-groups' of humpback whales at the South African and East Australian coasts^40,41^. Large groups (> 15 individuals) or group feeding events have not been described for fin whales anywhere else in the world. Published observations of fin whale feeding events comprised maximum group sizes of 13 animals^42,43^. Anecdotal reports from the nineteenth century suggest that, prior to their exploitation, fin whales used to aggregate at their feeding grounds in a similar way^44,45^. But for the post-whaling period our observed group sizes and the aggregative behaviour are novel. The footage presented in this study is the first documentation of large feeding aggregations of fin whales. It was featured in the 2019 BBC nature documentary ‘Seven Worlds, One Planet’, narrated by Sir David Attenborough, who notes the event as *'the largest congregation of great whales ever filmed*'. It has been suggested that the recovery to pre-exploitation numbers allows the re-emergence of behaviours, that, due to extremely low population numbers, had no longer been performed or observed^41,46^.

Lunge feeding, the dominant behaviour observed in feeding aggregations, is of particularly high energetic cost^47–49^. It is usually performed in areas of high prey density in order to make feeding efficient^47^. Baleen whales are thought to respond to prey distribution according to both aggregative and feeding thresholds. Aggregative behaviour then is responsive to local prey supply, and feeding occurs above a prey density threshold set by the energetic costs for lunge feeding^50^. The observations of large aggregations of actively feeding fin whales suggest both criteria to be met around Elephant Island.

Fin whale density was generally high throughout the surveyed area, but unless observed in aggregations, group sizes of fin whales were small (i.e., 1–4 individuals). The observed large feeding events were likely "sparked" by krill occurrences above a certain threshold density, triggering feeding and hence, concentrating fin whales from the wider area in one spot. The mechanism of attraction to the same prey cloud can currently only be speculated about. Maybe, the onset of feeding of a few animals attracts other animals in proximity, a phenomenon called 'local enhancement', often observed in birds, leading to a spontaneous occurrence of a feeding frenzy^51^. The cues to which fin whales respond to, i.e., visual, acoustic or other factors related to prey concentration however, are not yet known.

### Density and distribution

The density of fin whales in the survey area (0.0852 individuals/km^2^; 95% CI 0.0113–0.1696) was high for a large marine animal^52^ and particularly high compared to fin whale densities in other areas of the world, that are well known for fin whale occurrence (e.g. Southern California^53^, West Greenland^54^, Mediterranean Sea^55,56^). Within the survey area, fin whales were not evenly distributed, but concentrated in three areas along the shelf break west of the Antarctic Peninsula and particularly in a hotspot around Elephant Island, where an average density of 0.2123 (0.0521–0.383) individuals/km^2^ was predicted (Fig. 7). Similarly high densities have been estimated during a previous aerial survey around the South Shetland Islands in 2013 (0.117; 95% CI 0.053–0.181 individuals/km^2^)^15^, spatially matching one of the other hotspot areas. Although no records of feeding aggregations or large groups were included in the abundance survey data, the identified hotspots represent the areas where feeding aggregations have been observed in this study and previously^13,15^. These consistencies suggest that high fin whale densities and feeding aggregations are a recurring event at these hotspots. At the same time, the fact that no aggregations were observed during helicopter survey effort supports that the aggregations are spontaneous events lasting for short periods of time only, with animals engaging in a 'feeding frenzy' and then dispersing again to the nearer surroundings. During a dedicated aerial survey, the transects are covered at a survey speed of 80–90 knots, providing very little time for observation of any point along the transect and as such representing a snapshot of animal distribution. Chances to record rare events are limited and, from a methodological point of view, even undesired^19^. Our aerial survey captured the generally high densities of fin whales in the area without violating basic principles of distance sampling by including observations of extremely large groups. We suggest that the high animal density in the area forms the basis for spontaneous formations of feeding aggregations. These were detected from the ships in our study, which had considerably more observation time and actively searched for aggregations.

**Figure 8:**
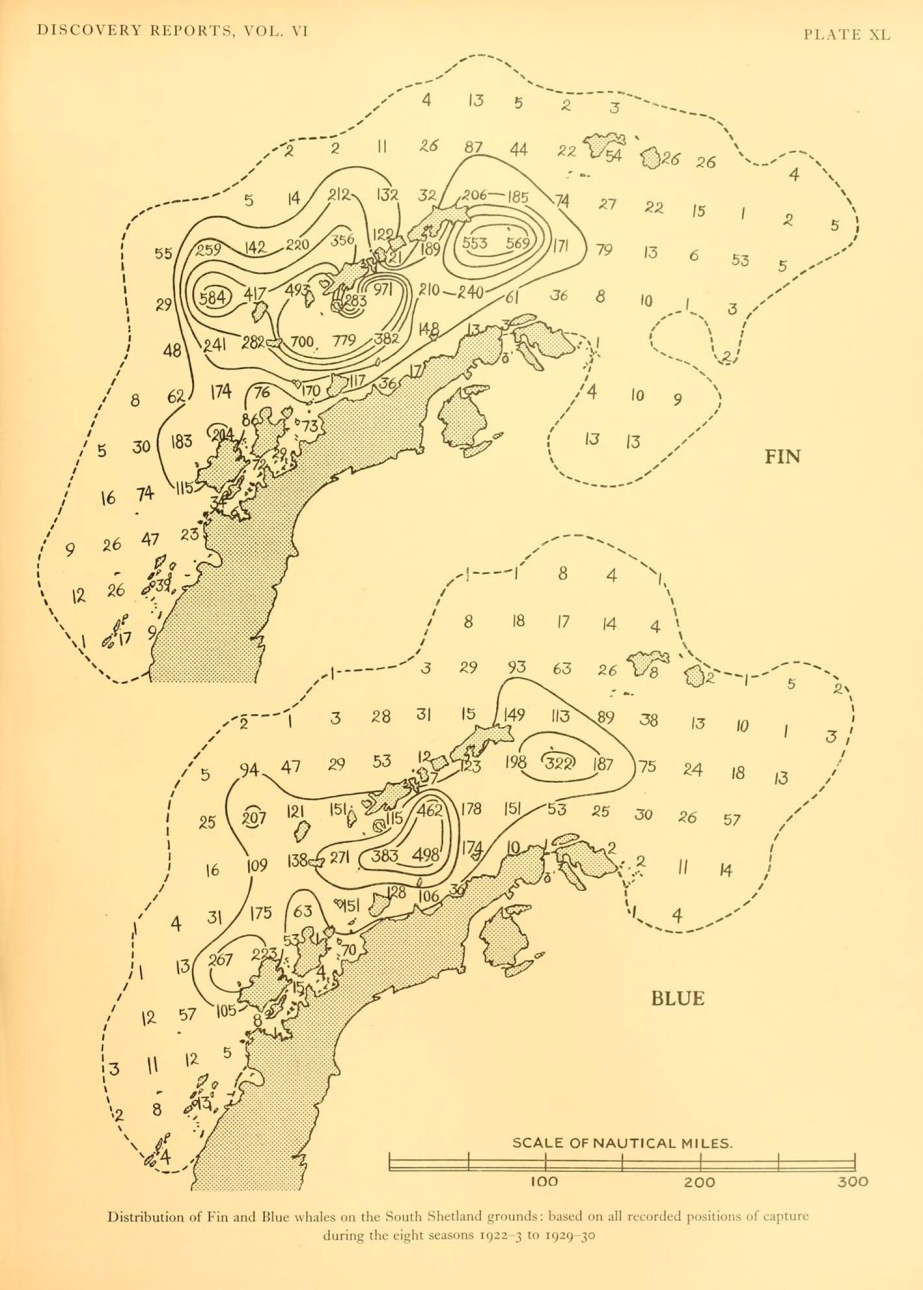
Original map from Kemp and Bennett (1932), showing the distribution of catches of blue and fin whales around the Western Antarctic Peninsula for the decade of the 1920s. It is apparent that historically, fin whales ranged inshore and in the Gerlache and Bransfield Strait in high numbers.

The hotspots identified in our study serve as feeding grounds for fin whales today. Catch records from the industrial whaling period identify the area around the tip of the Antarctic Peninsula as a major whaling ground, where large numbers of fin whales were caught at the beginning of the twentieth century (Fig. 8)^7^. In that region, the whalers targeted particular areas where they knew fin whales gathered for feeding, and beyond which it was considered needless to look for more once the whales were depleted^45^. Therefore, the catch records are a good reference for the location of historical feeding grounds. Our results support that fin whales have now returned to at least some of their ancestral feeding grounds.

Culturally inherited site fidelity to feeding and wintering grounds, transmitted through maternally directed learning and fidelity to important habitats^57–59^, is evident in many whale species^57,60–63^. The cultural knowledge of habitat as feeding grounds may be lost as a result of extreme population depletion^57^ and rediscovery is generally very slow, if it happens at all^59^. Blue whales *(Balaenoptera musculus)* around South Georgia had been assumed to have been depleted beyond a point of recovery^57^ and had disappeared completely from their former feeding grounds. Five decades later, increasing numbers of sightings and acoustic records have indicated a return of blue whales to their ancestral South Georgian foraging grounds, suggesting a rediscovery^64^. A combination of increasing population numbers and a rediscovery of important habitat, including transmission of this knowledge among the growing population, likely is the cause for large numbers of fin whales using their ancestral feeding grounds again. The fact that it has taken decades since the end of whaling until feeding aggregations of fin whales were observed again for the first time is indicative of the level of depletion and its spatial extent, leaving behind too few mature individuals for a swift recovery and re-occupancy of the habitat^64^. On a finer scale, some differences in the distribution of fin whales on the feeding grounds is apparent when comparing observed densities to historical catch records. The hotspot area around Elephant Island identified in our study (Fig. 7) matches the area from which feeding aggregations and observations of large numbers of fin whales have repeatedly been reported^13,14,16,65^, indicating some level of site-fidelity in recent times. However, this area is not reflected by particularly high catch records from the whaling period. This could indicate that fin whales did not concentrate in that particular area during whaling times. However, the discrepancy could also arise from the allocation of catch effort. Elephant Island is located at the outer limits of the former whaling ground and was visited much less by the whalers than the area around the South Shetland Islands, where whaling stations and ports were based. Distant areas of the whaling grounds were only visited on *'[…] exceptional occasions, when the whales were unusually scarce around the South Shetland Islands'*^7^. Consequently, low catch records around Elephant Island do not mean that historically fin whales must have been less abundant there.

On the other hand, catch records report highest fin whale catch-numbers around the South Shetland Islands and in the inshore areas of the Bransfield Strait (Fig. 8)^7^, indicating that fin whale numbers in these areas must have been high. While fin whales seem to have returned to the South Shetland Islands in large numbers, abundance in the Bransfield Strait remains low (this study^15,66,67^). Today, the inshore waters of the Antarctic Peninsula, including the Bransfield Strait, are dominated by humpback whales (*Megaptera novaeangliae*)^15,67–70^. Humpback whales have recovered from depletion by whaling at a much faster rate than other baleen whale populations in the Southern Hemisphere^71^. It is possible, that humpback whale dominance has caused fin whales to not reclaim their former feeding grounds in inshore waters, but predominantly using the outer shelf area. This horizontal niche-partitioning of the two species has been suggested previously by Herr et al.^15^. Another explanation may be that fin whale numbers are still small in comparison to historical times and expansion into ancestral feeding grounds is not yet complete.

### Population recovery

The results of our survey represent a snapshot of the minimum number (i.e., uncorrected for availability bias) of fin whales present in the area at the time of the survey. Comparing abundances between survey areas of different size and particularly with arbitrary choices of survey boundaries with regard to animal distribution and population boundaries, is mostly unfavourable. Therefore, density is the key metric for comparisons in time and space, also for comparison of our results with future estimates. However, the abundance estimate of 7909 (95% CI 1047–15,743) individuals suggests a considerable number of fin whales gathering again today in a comparatively small area off the Antarctic Peninsula during austral summer feeding season. In 2000, only 4672 (CV 42.37) whales were estimated fora much larger area comprising the Antarctic Peninsula and Scotia Arc region^9^. To put these numbers into a broader context, information on population structure, location of breeding grounds and migratory movements are needed. It remains unknown where these animals migrate from and if they belong to one or more breeding populations. Fin whales occur in both, the South Pacific and the South Atlantic^72^, therefore both Oceans are candidate areas of origin for these fin whales. Mixing of breeding stocks at feeding grounds is known from humpback whales^73,74^. But the population structure of Southern Hemisphere fin whales is not yet understood^75–77^, and the locations of lower latitude breeding or wintering grounds are unknown^78^. In the absence of this knowledge, we cannot draw definite conclusions on population recovery. However, if we rule out large-scale shifts of prey as another possible explanation for changes in observed animal abundance, a rediscovery of important habitat by a recovering population remains the most likely explanation for a re-occurrence of high numbers of fin whales at their historic feeding grounds. The Antarctic Peninsula region is known as a highly productive marine area with abundant krill, which has been sustaining large populations of krill predators throughout time^79–81^. Despite a suggested long-term decline in the krill stock^82^ and interannual fluctuations of abundance^83^, krill has not seen large scale distributional changes over past decades^84^ and sufficient biomass would have been available at the feeding grounds any time^81^. Therefore, changes in distribution and abundance of krill or other prey are unlikely explanations for the re-appearance of large numbers of fin whales at their historical feeding grounds.

### Environmental implications

As top predators, whales are indicators for ecosystem health. They are important ecosystem engineers, contributing to the stability and resilience of the ecosystem^85^. Recent estimates of prey consumption suggest that pre-exploitation populations of baleen whales in the Southern Ocean must have consumed 430 million tonnes of krill annually^86^, i.e., twice the estimated total biomass of *E. superba* today^87^. These new estimates lend additional support to the concept that whales fertilised their own feeding grounds in the Southern Ocean by feeding on iron-rich krill and discharging iron-rich faecal plumes in the surface layer^88–91^. In a region where primary productivity is largely limited by iron availability, whales would thus have substantially enhanced phytoplankton growth, boosting food availability for krill^91,92^. Krill biomass would thus be highly linked to whale abundance, explaining why the predicted 'krill surplus'^93^ as an effect of the removal of Southern Ocean whales from the ecosystem never materialised^89^, but krill instead declined^82^. The recovery of baleen whales and their nutrient recycling services, known as "the whale-pump"^91^, could thus augment primary productivity and restore ecosystem functions lost during twentieth century whaling^85,86^. A recovering fin whale population may lead to an increase of Southern Ocean productivity through enhancing iron levels in the surface layer^88^. By stimulating primary production, whales act as a carbon sink in the Southern Ocean^85,94^. This is of particular relevance, since the Southern Ocean is a major component of the coupled ocean–atmosphere climate system^95^, crucial for atmospheric carbon regulation and the most important ocean region for the uptake of anthropogenic CO_2_^96,97^.

## Conclusion

High densities, re-establishment of historical behaviours and the return to ancestral feeding grounds are promising signs for a recovering population. The aggregations documented in this study resemble descriptions of observers from the pre-whaling period: *'Whales' backs and blasts were seen at close intervals quite near to the ship and from horizon to horizon […]'*^44^, raising hope that fin whales are on their way to pre-exploitation numbers. In times of climate change, biodiversity loss and species extinction, the recovery of a large whale population is not only a glimpse of hope; it is also likely to have a stimulating effect on primary production in the Southern Ocean, enhancing CO_2_ uptake and carbon sink capacities.

## Supplementary Information

Supplementary Information 1.

Supplementary Information 2.

Supplementary Video S1.

Supplementary Video S2.

Supplementary Video S3.

Supplementary Video S4.

## Data Availability

Scripts and data used in the analyses can be accessed at https://github.com/sviquerat/FinWhaleReturn.
