# Supplementary figures and images for: Return of large fin whale feeding aggregations to historical whaling grounds in the Southern Ocean
Source: Sci Rep. 2022 Jul 7;12:9458. doi: 10.1038/s41598-022-13798-7 (PMC9262878; doi:10.1038/s41598-022-13798-7)

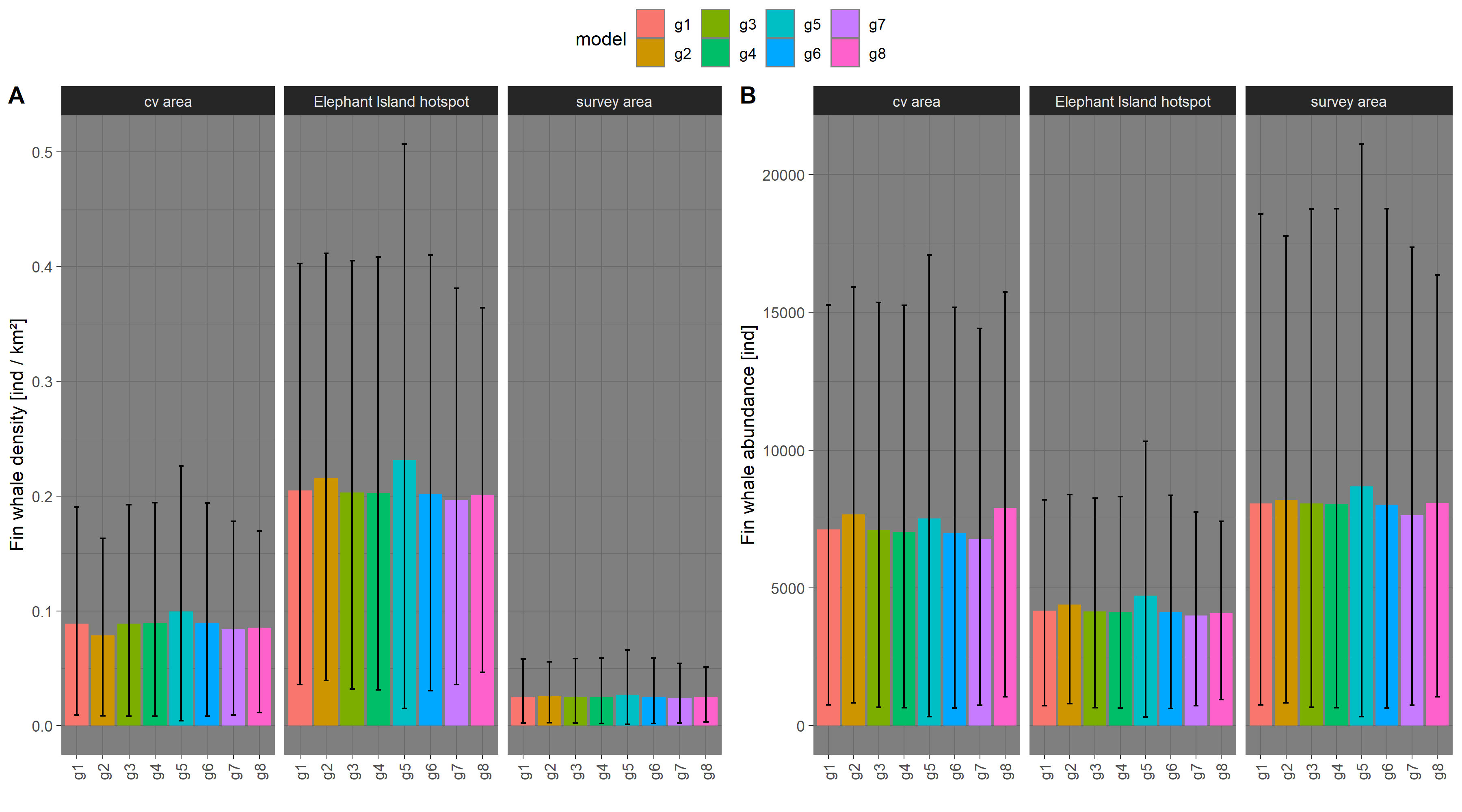

Supplement: Supplementary file 1 — Supplementary Information 1. [file 41598_2022_13798_MOESM1_ESM.png]
