# Supplementary material for: Return of large fin whale feeding aggregations to historical whaling grounds in the Southern Ocean
Source: Sci Rep. 2022 Jul 7;12:9458. doi: 10.1038/s41598-022-13798-7 (PMC9262878; doi:10.1038/s41598-022-13798-7)
Supplement: Supplementary file 2 — Supplementary Information 2. [file 41598_2022_13798_MOESM2_ESM.docx]

Online Supplementary Material

Figure captions

Figure S1

Boxplot showing abundance estimates (including 95% CI) predicted by all models tested. Model g8 was chosen based on lowest AIC and GCV scores.

Video Captions

Video 1: Video showing the high density of fin whales at the northern coast of Elephant Island in April 2018. The animals are spread out over the area and are currently not engaged in feeding.

Video 2: Aerial view of an active fin whale feeding aggregation filmed by drone in April 2018 at the northern coast of Elephant Island.

Video 3: Aerial close-up on an active fin whale feeding aggregation filmed at the northern coast of Elephant Island in April 2018.

Video 4: A distant view on an active feeding aggregation of approximately 150 fin whales filmed at the north coast of Elephant Island in March 2019. The horizon is covered in blows.
